# Care with child development and André Bullinger’s special look at prematurity

**DOI:** 10.1590/1984-0462/2023/41/2022208

**Published:** 2023-05-15

**Authors:** Jacques Sizun, Pierre Kuhn, Charlotte Tscherning

**Affiliations:** a University Hospital Toulouse, France.; b University Hospital, Strasbourg, France.; c Division of Neonatology, Sidra Medicine, Weill-Cornell Medical College, Doha, Qatar.

To the Editor,

We read with great interest the article “Care with child development and André Bullinger’s special look at prematurity” published in one of the latest issues of the Revista Paulista de Pediatria^1^. Authors rightly highlighted the impact of early environment on preterm infants’ neurobehavioral development and the need for early intervention aimed at optimizing this development. Additionally, they highlighted the importance of parental presence in the NICU.

The article is presented as a review of the literature in the PubMed, SciELO and Cairn databases. Unfortunately, the design of this review is not described, in particular the criteria for selecting articles, data extraction and synthesis of the results. This absence of a rigorous method explains the authors’ conclusions: “The Bullinger Approach (shows) … promising results for the prevention of neurodevelopmental disabilities, especially those related to orality”. The claim that the practice of “this approach can prevent neuromotor, language, eating and parent-infant relationship disorders in preterm infants” is not scientifically demonstrated and is pure speculation. No randomized clinical trial has been conducted to assess the impact of the Bullinger Approach. The specific techniques used in this program (asymmetrical positioning, contrasting checkerboard visual stimulation) have not been assessed for their impact.

Conversely, the other two programs described in this review are based on robust scientific data.

According to meta-analyses, the Kangaroo Mother Care is associated with an increase in breastfeeding rate in preterm babies^2^, a decrease in length of hospital stay^3^, and a reduction in mortality mainly in resource-limited settings^3^. Additionally, a randomized controlled study demonstrated a positive impact at adult age on IQ and attention scores^4^.

Meta-analyses demonstrated that the Newborn Individualized Developmental Care and Assessment Program (NIDCAP) is effective in improving preterm infants’ neurobehavioral and neurological development at two weeks of Corrected Age (CA)^5^, significantly reduces the length of hospital stay^6^, and increases the psychomotor development index at 9- and 12-months CA^6^. A randomized controlled study showed promising results at school age^7^. The large preterm birth cohort study (in France, 2011) Epipage 2 demonstrated that NIDCAP implementation significantly influenced the Kangaroo Mother Care initiation during the first week of age in preterm newborns compared with no training or with Bullinger Approach^8^.

Other early intervention programs such as Close Collaboration with parents^9^ or Family Integrated Care^10^ have been thoroughly evaluated and are not cited in this review emphasizing its lack of completeness.

As for medical treatments, the early non-pharmacological interventions in high-risk newborns need to be based on an evidence-based approach. This is important for the choice of the interventions, for the training of professionals as well as for information to parents.

We strongly recommend that randomized trials be conducted to assess the impact of the Bullinger Approach.
