# Supplementary material for: Care with child development and André Bullinger’s special look at prematurity
Source: Rev Paul Pediatr. 2023 May 15;41:e2022208. doi: 10.1590/1984-0462/2023/41/2022208 (PMC10184996; doi:10.1590/1984-0462/2023/41/2022208)
Supplement: Related texts

# Reply to letter to the editor

**DOI:** 10.1590/1984-0462/2023/41/2022208

**Authors:** Mônica Regina da Silva Raiol, Sylvie Viaux Savelon, Marcia Maria dos Santos de Moraes

**Affiliations:** a Universidade Paris 7, Paris, France.; b Pitié Salpêtrière, Paris, France.; c Universidade Federal do Sul da Bahia, Teixeira de Freitas, BA, Brazil.

The article “Care with child development and André Bullinger’s special look at prematurity” is very clear in its objective of “Addressing different forms of care for the development of premature children and their families in neonatal units, with emphasis on studies by André Bullinger”. Therefore, the statement that “this approach can prevent neuromotor, language, eating and parent-infant relationship disorders in preterm infants” cannot be considered “speculation”, since it was widely referenced in the works of André Bullinger and collaborators.

The article was accepted by this journal in the “review” category because, despite not having the traditional and internationally known systematic review format, it brings a compilation of works related to each of the forms of care for the development of premature infants, with special emphasis on the Bullinger approach.

Furthermore, articles with systemic and phenomenological approaches are still disregarded by technical sciences. Therefore, Bullinger, who was a sociointeractionist, a disciple of Piaget, is part of the currents inspired by experience, by dialectical material and, therefore, does not fit into the logic of evidence-based sciences. The article aimed to disseminate this author’s theories, which are still little known in our domain, with the aim of adding a new perspective to the care for the development of preterm infants and the deleterious effects of prematurity at medium and long terms.

As for the need for randomized studies, this is certainly an excellent proposal, as described at the end of the article: “This review also points to the need for further research on the subject and studies with a prospective-longitudinal design that could benefit newborns, particularly preterm infants”. Therefore, knowing the theory of the Bullinger approach, presented in this review, stimulates the performance of further comparative, prospective, perhaps multicentric studies that can add to the body of evidence on this approach relating to different aspects of the development of preterm infants.
